# Postmortem Studies of Fetal Grafts in Parkinson’s Disease: What Lessons Have We Learned?

**DOI:** 10.3389/fcell.2021.666675

**Published:** 2021-05-13

**Authors:** Jia-Yi Li, Wen Li

**Affiliations:** ^1^Laboratory of Neurodegenerative Diseases and Repair, Institute of Health Sciences, China Medical University, Shenyang, China; ^2^Neural Plasticity and Repair Unit, Wallenberg Neuroscience Centre, Department of Experimental Medical Science, Lund University, Lund, Sweden

**Keywords:** Parkinson’s disease, grafts, Lewy pathology, postmortem studies, transplantation

## Abstract

Neural transplantation is a potential therapeutic method for Parkinson’s disease (PD). Fetal dopaminergic (DA) neurons have been important transplantation cell sources in the history of replacement therapy for PD. Several decades of preclinical animal experiments and clinical trials using fetal DA neuron transplantation in PD therapy have shown not only promising results but also problems. In order to reveal possible factors influencing the clinical outcomes, we reviewed fetal DA neuron transplantation therapies from 1970s to present, with a special focus on postmortem studies. Firstly, we gave a general description of the clinical outcomes and neuroanatomy of grafted cases; secondly, we summarized the main available postmortem studies, including the cell survival, reinnervation, and pathology development. In the end, we further discussed the link between function and structure of the grafts, seeking for the possible factors contributing to a functional graft. With our review, we hope to provide references for future transplantation trials from a histological point of view.

## Introduction

Parkinson’s disease (PD) is characterized by the loss of dopaminergic (DA) neurons in the substantia nigra pars compacta (SNpc) of the midbrain. Replacement of the degenerated neurons has been thought to be a promising alternative approach for treating PD. Researchers have studied various cell and tissue types as sources for transplantation; for example, the adrenal medullary tissues from the patients themselves were once used for restoring nigrostriatal function. The method minimized possible immunological problems that might occur postoperatively. In 1982–1985, four patients with severe PD were subjected to an autologous adrenal medulla grafting procedure. Motor improvements were observed in one of the patients for 6 months after grafting (Backlund et al., 1985). Madrazo et al. (1987) grafted two patients with adrenal medullary tissue using a modified microsurgery technique in 1987, bringing the trials using this therapeutic method to a worldwide scale. As more trials were carried out, problems with transplanting adrenal medullary tissue began to appear, with unreproducible results, unsustainable outcomes, and severe side-effects (Goetz et al., 1989; Stoddard et al., 1989). Researchers were again in the urgent need for a promising cell source for PD transplantation therapy.

In the late 1970s, animal experiments demonstrated that the intracerebral grafting of rat fetal DA cells reversed the disease symptoms induced by 6-hydroxydopamine (6-OHDA) lesions (Bjorklund and Stenevi, 1979; Perlow et al., 1979). As one of the main pathological changes in PD is the loss of DA neurons in the SNpc, the method of using healthy cells to directly restore the decreased dopamine supply targeted the disease on a substantial level (Kalia and Lang, 2015). Repeated preclinical trials have been carried out since then, marking the beginning of using fetal DA neurons as cell source for transplantation to treat PD and giving hopes for the possibility of DA neuron grafting in PD patients (Brundin et al., 1986, 1987). It took nearly a decade before fetal DA neuron transplantation was brought to a clinical trial stage from the lab bench (Lindvall et al., 1988). Lindvall et al. (1988) reported a two patients’ 6-month follow-up postsurgery. With no complications shown after surgery, both patients exhibited minor improvements in motor performance accompanied by no striking change in response to drug treatment. However, one of the patients showed significant restored dopamine synthesis and storage in the grafted area, later revealed by 6-L-(18F) fluorodopa (FDOPA) scan (Lindvall et al., 1990). From the late 1980s, clinical follow-ups and postmortem studies on fetal DA transplantation studies in humans have been reported continuously, with diversity in clinical outcomes, sustainability of graft-induced benefits (GIB), survival of the grafts, pathology in grafted neurons, etc., hindering the clinical application of the method. Therefore, a systematic summary and analysis of the lessons we have learned from previous trials is necessary as references to improve the future transplantation therapy in PD. In this review, we have summarized the neuroanatomic studies of previous cases in relation to the clinical follow-ups, focusing on the DA neuron survival, reinnervation and pathology developments, analyzing the possible structure-function interplay of the grafted cells, and innovatively, from a thorough histological point of view, trying to reveal the possible factors contributing to a successful graft.

## General Description of Case Clinical Follow-Ups and Histology

Clinical trials using fetal DA neurons to treat PD have been continuing since the late 1980s. At the beginning of the 2000s, over 350 patients had been grafted with fetal DA tissues, including both single non-blind cases and double-blind placebo trials (Lindvall and Bjorklund, 2004; Bjorklund and Kordower, 2013). From the follow-up of these studies, promising improvements have been observed in some patients with respect to the following points: (1) improved motor function with speeded up movement, reduced rigidity, and decreased time in “off” periods; (2) maintenance of daily life quality with a reduction in levodopa (L-dopa) treatment; and (3) survival and functionality of grafted tissues observed through FDOPA uptake positron emission tomography (PET) scan. We included in this review (Hagell and Brundin, 2001) studies with detailed description of postmortem results, to reveal the possible relations between neuroanatomy and symptomatic improvements.

In 1990s, autopsy reports with transplanted patients who died shortly after surgery were already reported. Redmond et al. (1990) reported one case grafted at the age of 63, with the patient dying 4 months after transplantation. No major clinical improvements were observed. Postmortem analysis showed no survival of tyrosine hydroxylase-positive (TH+) neurons (Spencer et al., 1992), possibly due to the short survival time postgrafting. The following year, a study with the clinical follow-up of 12 patients showed promising improvements in motor performance with a decreased demand for L-dopa treatment and reduced time spent in the “off” period, in most patients 12 months after operation. Among these patients, five died between 18 and 40 months postsurgery (Henderson et al., 1991). Three of them exhibited TH+ neurons in the graft-host interface, in which some cells showed complete TH staining while others contained neuromelanin (NM). However, there was no clear sign of graft innervation (Henderson et al., 1991). Kordower et al. (1995, 1996) performed a detailed autopsy study on one of these patients, who died 18 months posttransplantation. The patient showed great clinical improvement and striatal graft FDOPA uptake reached 72% of normal levels, 15 months after surgery. Histological analysis presented a massive quantity of cell survival bilaterally with fiber extension into the host putamen (Kordower et al., 1995, 1996). In 1995, a female patient who received grafts at the age of 61 was reported to have benefited greatly from the transplantation with a significant motor functional recovery during the first 12 months postsurgery. She died 19 months postgrafting. Histopathology showed over 138,000 TH+ cells in grafts without any detection of NM. The graft reinnervated 78% of the host putamen. No Lewy pathology was detected in the graft (Freeman et al., 1995). Among the above cases, promising clinical benefits were already present approximately 1-year postoperation, with positive correlation toward the TH+ neuron survival in the grafts.

In the 2000s, fetal neuron transplantation entered a new age with the emergence of double-blind clinical trials. In 2001, a double-blind transplantation study including 40 patients with graft and sham was reported (Freed et al., 2001). In the grafted group, three patients died before the end of study at 30 days, 7 months, and 3 years postoperations, in which the 3-year-old graft induced significant motor improvement for the patient, with a decreased Unified Parkinson’s Disease Rating Scale (UPDRS) score and 100% restored FDOPA uptake. All three cases showed numerous amounts of TH+ cells and degenerating NM containing cells in the grafts. TH+ fiber outgrowth was observed from the grafts, particularly seen in the 3-year case, which had extensive fiber reinnervation into the whole putamen (Hagell and Brundin, 2001). In 2005, two autopsy studies were reported by Isacson et al. (Mendez et al., 2000). In both studies, the patients survived longer than 3 years with marked clinical improvements, showing 30–40% decreased UPDRS score and a significant increase in FDOPA uptake during long-term follow-up (Mendez et al., 2002, 2005; Hallett et al., 2014). In the postmortem analysis, the grafts maintained a large number of TH+ neurons with NM present. Extensive reinnervation was observed spreading out from the grafted neurons. Markedly, functional profile of the TH+ neurons was observed with dopamine transporter (DAT) positive staining (Mendez et al., 2002, 2005; Hallett et al., 2014). In these cases, with the rather “young” grafts (7 months to 3 years), no graft containing Lewy pathology had been observed.

In 2008, three groups reported their work on the survival of grafted neurons 12–16 years postsurgery (Lindvall et al., 1990, 1992, 1994; Wenning et al., 1997; Hagell et al., 1999; Hauser et al., 1999; Mendez et al., 2002; Olanow et al., 2003). All three cases showed significant GIBs at the early stages, particularly within the first 10 years, with massively shortened “off” period time and significantly reduced UPDRS scores. Postmortem studies revealed large amounts of survived grafted neurons (Mendez et al., 2005, 2008; Kordower et al., 2008a, b; Li et al., 2008; Chu and Kordower, 2010; Hallett et al., 2014). In 2011, our group reported the postmortem analysis of a patient who received fetal DA neurons 22 years prior to death. The graft did not show clear benefit to the patient, with very mild symptom relief and no change in FDOPA uptake (Lindvall et al., 1988). Only 2,700 neurons were observed in one injection tract, and almost no reinnervation was detected (Kurowska et al., 2011). In 2016, a 24-year case was reported, being the longest surviving case posttransplantation at the time. Unilateral grafts in the right putamen contributed major benefits to the patient in the first 14 years following the surgery. The patient performed well without “off” periods or L-dopa treatment for several years, yet, in the later stage before his death, the condition worsened (Lindvall et al., 1990, 1992, 1994; Wenning et al., 1997; Piccini et al., 1999). Autopsy assessments exhibited a considerable number of survived DA neurons and extensive reinnervation throughout the entire grafted putamen, which correlated to the major improvement in the FDOPA uptake test in the clinical follow-up (Li et al., 2016). In the following year, Kordower et al. (2017) reported a case with 16-year-old grafts, presenting no significant motor improvement; however, large profiles of grafted neurons were shown accompanied by extensive reinnervation into the host putamen. Interestingly, massive Lewy pathology profile was detected in grafted neurons (Olanow et al., 2003; Kordower et al., 2017). Although, survival and reinnervation were assuring in most of these long-term (more than 10 years) cases, GIBs were shown to be absent or unsustainable. Nevertheless, almost all these long-survival grafts showed Lewy pathology development in different extents in the grafted cells, suggesting a possible role of graft Lewy pathology in affecting the graft function and clinical outcomes.

Summary of clinical follow-ups and autopsy reports are shown in Supplementary Tables 1, 3.

## Summary of the Autopsy Studies From the Grafts

### Grafted Neuron Survival

From the cases summarized above, we concluded four aspects to describe the survival of the grafted neurons: (1) time: graft ages ranged from 30 days (Freed et al., 2001; Hagell and Brundin, 2001) to 24 years (Li et al., 2016). TH+-grafted neurons were observed in grafts of 30 days already. Moreover, the 24-year-old case which Li et al. (2016) reported proved that transplanted neurons can survive for long period of time up to a quarter of a century; (2) number: besides the cases where the number of TH+ neurons were not reported (Mendez et al., 2008), the population of survived neurons were mostly from 12,000 (Li et al., 2008) to 330,000 (Kordower et al., 2017), showing large profiles of cell survival; (3) NM: some of the grafted neurons contained NM, with mild or no distinct TH staining, indicating degeneration of DA phenotypes; (4) not all reported autopsy cases showed TH+-grafted neurons, which were seen for example in a study reported by Henderson et al. (1991) where grafted cells in two of the patients failed to show any TH immunoreactivity (Hitchcock et al., 1994; Hagell and Brundin, 2001).

### Graft Reinnervation

In order to restore the denervated DA terminals, grafts with large amounts of survived TH+ neurons needed to reinnervate the host putamen or caudate nuclei. Therefore, the autopsy results of TH profile reinnervation were crucial to evaluate the functional aspects of the grafts. Among the cases summarized above, the extent of reinnervation varied. Based on the patterns and percentage of reinnervation, we ranked them into: (1) almost no integration: often shown as very few short fiber outgrowth from the edge of the grafts, such as the postmortem case reported by Kurowska et al. (2011), where the 22-year-old grafts did not integrate into the host striatum sufficiently, with sparsely distributed fibers reaching out from the grafted neurons; (2) partial reinnervation: often presented as fiber outgrowth for several millimeters (Kordower et al., 1997), which extended to parts of the host tissue, forming a patch/matrix pattern (Kordower et al., 2008a) or organotypic shape (Chu and Kordower, 2010); (3) extensive integration: dense TH+ long processes reached out to almost the full width of the host putamen or, in some cases, reinnervating the whole putamen. For example, in the 14-year-old grafts that Mendez et al. (2008) reported, reinnervation was detected throughout the putamen, with TH fiber density comparable with non-PD subjects.

### Graft Lewy Pathology

Alpha-synuclein (α-syn) aggregation, which forms the main parts of Lewy bodies (LBs), is one of the main pathological features of PD (Yates, 2019). Therefore, evaluating the Lewy pathology in grafted cells is important to understand the progress of the disease in PD patients’ brain. In the cases we summarized, grafts younger than 4 years did not show any LB formation in the grafted cells (Hallett et al., 2014). Although some of the grafted cells were present with increased level of soluble cytoplasmic α-syn, no aggregates were detected (Kordower et al., 2008a). On the contrary, in most of the grafts older than 10 years, grafted neurons contained certain percentages of LBs, ranging from 1.2 to 27%. The morphology of these LBs was undistinguishable from the host nigral aggregates (Kurowska et al., 2011; Kordower et al., 2017). In some cases, the graft LBs were also thioflavin S and ubiquitin positive (Kurowska et al., 2011). Random or abundant Lewy neurites (LNs) were also present occasionally (Li et al., 2016). Nevertheless, there are still ongoing transplantation cases where the patients are still alive to the point of this review. These cases have revealed even longer graft functioning time, such as the two cases reported by Kefalopoulou et al. (2014), where motor improvements sustained for 18 years by 2014, providing a “proof-of-concept” support for the effectiveness of fetal DA neuron transplantation therapy.

Summary of clinical follow-ups and autopsy reports are shown in Supplementary Tables 1, 3.

## Structure and Function Interplay of the Grafted Neurons

Postmortem studies presented us with the structural status of various DA grafts, including the survival of the grafts, the reinnervation of the grafted tissue, and the Lewy pathology development in grafted cells. How did these histological features reflect the functional outcomes of the grafts? From the summary and analysis of the autopsy cases above, we reasoned that: (1) sufficient survival and reinnervation of the grafted cells was the necessary foundation of functional grafts; (2) Lewy pathology of the grafted cells influenced the sustainability of the graft function.

### Grafted Cell Survival and Reinnervation Was Necessary for the Grafts to Function

#### Survived DA Neuron Number

First, how sufficiently the grafted neurons survived the transplantation was the basic requirement for them to function. In preclinical experiments, the graft survival and integration appeared to be highly relevant to the motor improvements (Nakao et al., 1994). In some clinical trials, FDOPA uptake, which indicated the cell survival and fiber density, was positively correlated with the symptom relief (Hagell et al., 1999; Hauser et al., 1999). The quality of graft survival was firstly reflected by the number of cells present in the transplants. As we discussed above, cell number was mostly above 12,000 in various grafts (Li et al., 2008). It is difficult to determine what the proper number of cells would be the threshold level for a graft to function. However, the presence of very few neurons may contribute to a failed transplantation. For example, a report with graft age ranging from 18 to 40 months showed significant motor improvements in the clinical follow-ups. Postmortem analysis revealed surviving DA neurons in the graft-host interface, partially of which showed intense TH staining (Henderson et al., 1991). On the contrary, in the case Kurowska et al. (2011) reported, few grafted cells were detected in one injection tract. Clinical follow-ups revealed very mild symptom relief correlatedly. Therefore, sufficient amounts of transplanted cells defined the qualifying level for a functional graft.

#### Functional Profile of the Grafted Neurons

Second, the presence of functional profiles of DA neurons, such as TH, DAT (Olanow et al., 2003; Kordower et al., 2008a; Chu and Kordower, 2010), G-protein-regulated inward-rectifier potassium channel 2 (Girk2) (Mendez et al., 2000, 2002, 2005; Hallett et al., 2014), etc., were also important for the comprehensive functionality of the grafts. From the cases shown, we observed that not all the transplanted cells were TH positive, with some only containing NM. In the trial Spencer et al. (1992) reported, the cells in the 4-month-old graft contained NM; however, no TH+ cells were found (Redmond et al., 1990). Clinical follow-up showed no sign of motor improvements. In the case Kurowska et al. (2011) reported, histological study showed approximately two-thirds of the grafted neurons exhibited TH immunoreactivity (over 27% only NM positive. The long-term clinical follow-up showed transient mild improvements (Lindvall et al., 1988). Similarly, in a case Li et al. (2008) reported in 2008, a large percentage of grafted neurons were shown to be only NM positive. Modest GIBs was observed from the bilateral grafts (Li et al., 2008). Besides TH, DAT immunoreactivity was also observed in some sustainably well-functioned grafts, such as the grafts reported by Mendez et al. (2008), where after 14 years, the graft function was well maintained with UPDRS score up to 50% decrease.

#### Graft Reinnervation

At last, the reinnervation of the grafts into the host tissue was the key to a beneficial transplantation. As discussed above, there were different degrees of integration from the grafts. Seemingly, there existed a positive correlation between graft survival and reinnervation with the functional recovery. Thorough and extensive integration, especially the ones reinnervated the whole brain region, often resulted in beneficial grafts, such as the cases reported by Kordower et al. (2008a), Li et al. (2016), and Mendez et al. (2008), where patients showed major improvements on motor performance, significantly decreased UPDRS scores and less L-dopa dosage requirements. In all the three cases, postmortem studies revealed dense network of DA profiles extending into the host striatum, forming extensive reinnervation. On the contrary, grafts with sparsely extended TH+ fibers or just reaching out to the surrounding putamen generated mild improvements or unsustainable effects, such as the cases reported by Li et al. (2008), where long processed neurons formed dense networks with the surrounding putamen. Motor improvements appeared to be mild in one of the patients, while in the other, the originally significant GIBs started decaying 3 years postsurgery.

In summary, there existed possible positive correlation between the graft survival and reinnervation with the functionality of the grafted cells and the following clinical outcomes, which the sufficient grafted cell number, functional profile such as DAT immunoreactivity and extensive reinnervation lays out the foundation for a successful graft. However, not all the grafts survived and reinnervated effectively showed GIBs. Among the cases summarized, it is noticeable that the case reported by Kordower et al. (2017) showed no clinical recovery over all, but exhibited large numbers of DA neurons surviving and reinnervating the host tissue. In some cases, grafted cells showed promising benefits in the early stages posttransplantation, however, gradually lost their function along time (Li et al., 2016). Therefore, there may exist factors counteracting to the benefit generated by the originally healthy grafted cells, leading to the decaying of GIBs.

### Grafted Cell Neuropathology Development Influenced the Sustainability of Graft Function

Lewy pathology with α-syn aggregation was found to be cellular toxic, by inducing multiple neuronal dysfunctions such as mitochondrial deficiency (Vekrellis et al., 2011; Fares et al., 2021). The development of Lewy pathology in the originally healthy grafted neurons might lead to their early degeneration, therefore causing the gradual loss of GIBs. This was suggested by the autopsy studies of several long-term cases. For example, the 14-year case Kordower et al. (2008a) reported and the 24-year case reported by our group (Li et al., 2016) both showed significant symptom relief in at least the first 10 years postsurgery, with less or even no L-dopa treatments needed. However, in the later stages, especially after 10 years postoperation, worsening of symptoms began to appear until the GIBs completely disappeared prior to death. Coincidentally, only grafts that were older than 10 years showed LBs in the grafted cells. In both cases, LBs were found in the grafted neurons. In our 24-year case, 12% of the grafted cells contain typical LBs, in which 11% were also ubiquitin immunoreacted (Kordower et al., 2008a; Li et al., 2016). Moreover, study by Kordower et al. (2017), the 16-year-old grafts showed robust survival and extensive reinnervation, however, exhibited 10.7 and 27% LB containing grafted cells bilaterally, contributing possibly to no motor improvements and mild symptom relief in the clinical follow-ups. The decaying and absence of GIBs in these long-term cases suggested that the pathology development in grafted neurons influence the function and sustainability of the grafts. Even a viable graft could lose its function or generate no benefits at all, if heavy loads of Lewy pathology affected the transplants.

### The Possible Role of Serotoninergic System

Graft-induced dyskinesia (GID) is defined as unvolunteered movements which appeared after transplantation and could not be eliminated during off-medication period (Winkler et al., 2005). In the transplantation trials, a considerable number of patients experienced GID. In the 34-patient double-blind trial by Olanow et al. (2003), 56% of patients experienced dyskinesia after withdraw of medication. Open-label trial with 14 patients that were followed up to 11 years also showed mild to moderate level of dyskinesia, which did not correlate with the FDOPA uptake (Hagell et al., 2002). Therefore, the appearance of GID was one of the complications affecting clinical outcomes.

There have been discussions about the mechanisms behind GID from a histological point of view; for example, a sustained effects of L-dopa treatments which made the patients more “dyskinetic” (Winkler et al., 2005). Barker and Kuan (2010) discussed the importance of non-dopamine system such as serotoninergic neurons in influencing the nigrostriatal performance. These neurons can convert, store, and release dopamine through nerve terminals. However, due to the lack of DAT (as seen in the postmortem results of several cases), inactivation of dopamine could not be completed (Tanaka et al., 1999). It was shown that in patients with GID, the serotoninergic neurons “hyper-innervated” the grafted striatum (Politis et al., 2010). Researches in humans showed also that serotonergic innervation promoted excessive release of dopamine in the striatum, which contributed to the formation of L-dopa-induced dyskinesia (Espay et al., 2018; Munoz et al., 2020). The accurate mechanism behind GID is still not clear. It may be more of a complicated network problem than a single mechanism induction. However, these still showed that the 5-hydroxy tryptamine (5-HT) cells may play an important role in causing GID, therefore, should be evaluated in the future anatomic studies.

## Lessons We Learned From the Autopsy Studies—How to Improve Transplantation Therapy

From the discussion above, we confirmed that there existed unneglectable relation between the graft anatomic features and the clinical outcomes. It was undeniable that the effects of cell replacement therapy were not yet optimal. It is necessary for us to analyze and summarize the reported cases and obtain reference on how to improve the fetal DA neuron transplantation.

### Improving the Survival and Reinnervation of the Grafted Cells

Brundin et al. (2000) reported a case of five patients grafted with cell suspension pretreated with lazaroid, a lipid peroxidation inhibitor. The trial managed to reach similar level of clinical symptom relief compared with cases without lazaroid addition reported by the same center previously, however, with a decreased amount of graft tissue needed for the transplantation (Brundin et al., 2000). Mendez et al. (2000) reported cases of patients grafted with cell suspension pretreated with glia cell line-derived neurotrophic factor (GDNF). In two of the patients, PET scan showed a mean uptake increase of 107%, suggesting the improvement of cell survival. In the clinical follow-up, both patients had decreased time in “off” phase, with UPDRS score significantly reduced (Mendez et al., 2000). Moreover, in the postmortem analysis of these GDNF-pretreated grafts, the functional profiles of the grafted cells were significantly improved with DAT and Tom20-positive cells present (Mendez et al., 2005; Hallett et al., 2014). It seemed that a positive value of using neurotrophic factors in pretreatment of grafted neurons was shown. Therefore, neurotrophic factor addition may be a potential method for improving the survival and long-term health of the grafted cells. However, the effects of using biomolecules such as GDNF must be investigated more thoroughly before applying to the transplantation therapy. The mechanism of neural protection from neurotrophic factors such as GDNF is the activation of cascades included in specific cell signaling pathway, which then promotes downstream effects such as neurite outgrowth and synaptic plasticity (Airaksinen and Saarma, 2002). Researchers have performed clinical trials using neurotrophic factors for treating PD, both by directly delivering into the brain (Lang et al., 2006) or genetically overexpressing through adeno-associated virus (AAV) vectors (Marks et al., 2010). In placebo-controlled trials, no significant improvements were seen in the primary endpoints (Olanow et al., 2015). Therefore, we should be cautious about drawing conclusions on the effects of pretreating grafted cells with neurotrophic factors.

The age, viability, quantity, and quality of grafted materials defined the start of transplantation trials, therefore possessed great importance in affecting the survival of grafted neurons (Bjorklund and Lindvall, 2017). In mouse experiments, fetal midbrain tissue obtained around 11–14 days of gestation were shown to yield a high number of TH+ neurons after transplantation, which indicated the narrow window of transplantable fetal tissue, which was the actively developing stages of A9 DA neurons, before the outgrowth of long neurites (Torres et al., 2008; Bye et al., 2012). Therefore, previous trials using fetuses at 6–9 weeks of gestational age had generally large profiles of survived neurons (Hallett et al., 2014; Li et al., 2016). Besides the age of grafted fetuses, the viability of the transplanted cells or tissues also played an important role in determining the efficacy of the grafts. In the trials summarized in Supplementary Table 2, both cell suspension and tissue blocks had been used in the preparation of grafted materials. It was suggested that the preparation of cell suspension may compromise the number of viable neurons for transplantation, that only 20% donor cells survived the preparation procedures (Karlsson et al., 2005). Moreover, the transplantation sites can also influence the graft survival (Redmond et al., 2008). From our summarized cases, the putamen grafts in general showed larger amounts of survived neurons and correspondingly better recovery from the patients (Mendez et al., 2005; Li et al., 2008).

### Inhibiting the Development of Graft Lewy Pathology

As discussed above, one of the key factors contributing to the loss of grafted neuron function is the development of Lewy pathology in the grafted neurons. Inhibiting the formation and propagation of α-syn aggregates could be a feasible way to sustain the GIBs.

#### Possible Factors Contributing to Graft Lewy Pathology Development

The development of Lewy pathology in the grafted neurons was possibly influenced by various factors. Firstly, α-syn aggregation in the surrounding or projecting host brain regions may propagate to the originally healthy grafted neurons. Therefore, more severe host brain pathology may also result in a heavier loading of LBs in the grafted cells. It has been reported that α-syn can spread from cell to cell (Hansen et al., 2011; Lee et al., 2012) and from the peripheral tissue to the brain (Braak et al., 2003; Goedert et al., 2010; Holmqvist et al., 2014), inducing multiple aspects of cell dysfunction (Schulz-Schaeffer, 2010; Winslow et al., 2010; Subramaniam and Chesselet, 2013; Wong and Krainc, 2017), such as dopamine release. In the 24-year case, we reported, severe Lewy pathology were detected in multiple regions in the host brain, which resulted in 12% of the grafted cells loaded with LBs (Li et al., 2016). In a recently published work by Hoban et al. (2020), a fraction of the DA neurons transplanted into a humanized rat model, obtained by coinjection of preformed human α-syn fibrils and AAV expressing human wild-type α-syn, developed LBs, suggesting the propagation of α-syn pathology from host to graft. Secondly, the presence of neuroinflammation can contribute to the LB formation in the grafts. In a study reported by Olanow et al. (2019), grafts ranging from 18 months to 16 years were analyzed in the aspects of microglia activation and α-syn aggregation in the grafts. Microglia activation was already present in the 18-month graft, while LB formation only started to appear in 14- and 16-year-old grafts, suggesting the temporally advanced deposition of neuroinflammation compared with Lewy pathology development, which suggested that microglial activation contributed to the development of α-syn pathology in the grafts (Olanow et al., 2019).

#### Inhibition of α-Syn Aggregation and Propagation

Knowing the factors contributing to α-syn aggregation in grafted neurons, how can we develop pathology-targeted approaches to improve the transplantation outcomes? First, we could seek for methods targeting the accumulation and aggregation process of α-syn from the grafted neuron side. α-Syn propagation follows a “prion”-like manner, where external α-syn seeds template the endogenous overexpressed or mutated α-syn monomers to form secondary aggregation (Goedert et al., 2017; Pineda and Burre, 2017). Therefore, modulating α-syn aggregation in grafted cells may decelerate the Lewy pathology formation. As the toxic species of are mainly the aggregated forms such as oligomers and fibrils (Lashuel et al., 2013; Fares et al., 2021), conformation-specific antibodies which binds to certain species of α-syn, therefore, inhibit the aggregation initiation and progression. Several antibody treatments have been proven effective (De Genst et al., 2010), some of which have already moved on to clinical trial phase, after proven effective in preventing formation of aggregates or deattenuate the misfolded α-syn (Krishnan et al., 2014). Besides strain-specific antibodies, small molecules and chemical compounds have also been proven effective in inhibiting α-syn aggregation. For example, the 3-(1,3-benzodioxol-5-yl)-5-(3-bromophenyl)-1H-pyrazole (anle138b), a compound binding protein aggregates specifically, showed capacity of inhibiting α-syn oligomer formation both *in vitro* and *in vivo* (Wagner et al., 2013). Additionally, protein homeostasis requires the effective clearance of misfolded and aggregated forms of proteins (Vilchez et al., 2014). Therefore, inducing an enhanced protein degradation system can be considered a potential preventing approach for α-syn pathology in the graft. Elevating patient protein degradation system in general may be hard to achieve and can cause side effects, while targeting specifically grafted cells may be feasible. Many approaches can be employed to modulate the protein metabolism, for example, regionally administrating autophagy and lysosome enhancer such as rapamycin, which targets mTOR (Waldner et al., 2016; Moors et al., 2017). Our previous work also demonstrated the effect of bioactive herbal extracts, dihydromerycetin (DHM) and salvianolic acid B (SalB), in inhibiting α-syn aggregation by augmenting chaperone mediated autophagy (CMA) (Wu et al., 2019). However, one should be cautious on excessively reducing α-syn, as the protein plays important physiological roles in neuronal synaptic function. Ninety percent loss of α-syn can induce a significant neuronal toxicity as reported by Gorbatyuk et al. (2010). Native α-syn was also shown to have an immune protective role against RNA viral infections in the central nervous system (CNS) (Beatman et al., 2015). Silencing α-syn expression in the nigrostriatal system resulted in neuronal dysfunction, which sequentially triggered neuroinflammation (Benskey et al., 2018). Therefore, finding a method that can fine tune α-syn level which exhibits a sensitive response to α-syn-level alteration can be the future direction of efforts.

#### Possible Role of Inhibiting Inflammation

Neuroinflammation plays a crucial role in affecting α-syn aggregation in the grafted cells, as discussed above. Therefore, one could reason that the use of immunosuppression may influence the pathology development in the grafts. As the immunosuppression process may require long-term and frequent medical involvements, which could cause risk of postsurgery complications, there were researchers choosing not to apply immunosuppression. For example, in the double-blind study by Freed et al. (2001) involving 40 patients, none of the patients received immunosuppression. In the cases Lund University researchers reported, patients received long-term triple cocktail immunosuppression similar to that used in organ transplantation (Muntean and Lucan, 2013). In the double-blind trial reported by Olanow et al. (2003), the researchers picked a middle ground using cyclosporine treatments for a short period of time (6 months). The clinical outcomes of these cases failed to indicate a clear advantage of immunosuppression. However, the absence of immunosuppression may preserve a long-term effect of causing neuroinflammatory responses, which may induce malfunction of the grafted tissues. In the postmortem cases, Kordower et al. (2008a) reported from 2008 to 2009, where no immunosuppression was given, CD45-positive microglia were shown to be abundant in grafted regions (Kordower et al., 2008a; Chu and Kordower, 2010). On the contrary, the 2016 case we reported, where triple-cocktail immunosuppression was used, showed no significant activation of microglia comparing with normal subjects (Li et al., 2016). Therefore, the application of immunosuppression may present an antagonym effect toward long-term neuroinflammation and protect the grafted neurons against excessive Lewy pathology. It has been suggested that solid, non-dissociated pieces of grafting tissues, compared with cell suspension, have higher potential to trigger inflammation at the grafted sites, which may in the end result in large amounts of graft Lewy pathology and reduced graft efficacy (Kordower et al., 2008a). The remaining donor blood vessels in non-dissociated tissues, which expressed major histocompatibility complexes class I (MHC I), could trigger the immune reaction in the recipient brains (Geny et al., 1994). It was proven in non-human primate study that solid pieces of tissues induced a stronger inflammation compared with cell suspensions (Redmond et al., 2008).

### Other Factors Influencing the Clinical Outcomes of Cell Replacement Therapy

#### Patient Selection Before Entering Transplantation Trials

Other than improving the survival and reinnervation of the grafted neurons and inhibiting their LB formation, there are certainly many other factors influencing the structure of the grafts and the consequential clinical outcomes, for example, the integrity of the host nigrostriatal pathways. Reinnervation of the grafts into the host striatum was the key to a successfully functional graft; therefore, the host nigrostriatal system should at least be partially present. In animal experiments, it has been shown that the effects of intrastriatal grafts depend on the degeneration severity of the host nigrostriatal pathways (Kirik et al., 2001). A reliable host nigrostriatal system is important for the graft to reach out and re-establish the neuronal network. The disease severity of PD patients can influence the extent of graft innervation, which raised the questions of whether patients should be selected based on the progression of their disease symptoms and even pathology severity in the brain (Piccini et al., 2005; Barker et al., 2013, 2015). Meanwhile, previous open-label cases have obtained promising results in some patients as discussed above. However, the two double-blind placebo-controlled failed to show a statistically significant difference between the grafted group and sham (Freed et al., 2001; Olanow et al., 2003). In one of the double-blind studies, it was observed that the transplantation benefited younger but not older patients (Freed et al., 2001; Ma et al., 2010). Based on these reasoning’s, the TRANSEURO consortium was initiated. After summarizing discussion of main investigators involved in previous trials, it was suspected that DA neuron transplantation to earlier stage or less severe PD patients may result in possibly greater recovery post-surgery (Barker et al., 2015). The TRANSEURO program therefore selected younger and early-stage PD patients from different countries and put them into observational cohorts. Following standardized protocols of clinical follow-ups and surgery, the program has now been going on for 10 years. Besides the confirmation that earlier-stage PD patients were more suited for transplantation study, the program has also provided substantial implications and recommendations, such as an established patient-selection criteria and standard operation procedures, for future cell replacement therapy overall, including stem cell-based transplantation (Barker and Consortium, 2019).

#### Pluripotent Cell-Based Transplantation: New Era of Cell Replacement Therapy in PD

Fetal DA neuron transplantation had its limitation which could not be overcome by methodological improvements, such as the supply of fetal tissue and related ethical problems. Therefore, cell replacement therapy needed a new source of grafting cells to move forward and become clinically scalable. Stem cell-based transplantation therefore came to the forefront. Stem cells such as human embryonic stem cells (hESCs) and human-induced pluripotent cells (hiPSCs) are theoretically unlimited cell sources due to their capabilities to proliferate, therefore eliminating the issues regarding tissue acquiring with fetal cells (Thomson et al., 1998; Takahashi et al., 2007). Their embryonic status gives them the potential of differentiating into different types of adult somatic cell, including DA neurons and neural progenitors. Preclinical experiments have involved both hESCs and hiPSCs (Grealish et al., 2014; Kikuchi et al., 2017; Song et al., 2020; Tao et al., 2021). Compared with hESCs, which are the standards for pluripotent cells, hiPSCs induced concerns previously as their generation process included genetic modification (Li et al., 2015). Even with the optimized reprogramming protocol without any use of viruses, hiPSCs still possess genetic and epigenetic risks in the long run. Therefore, some researchers have chosen hESCs as cell sources for implantation, such as the STEM-PD program (Kirkeby et al., 2017). However, hESCs still bring with them the ethical concerns like fetal DA cells. Moreover, the necessity for immune suppression and the risk of neuroinflammation still remain.

Human-induced pluripotent cells give the possibility of performing autologous transplantation, as they can be derived from the patients’ own or HLA-matched individuals’ somatic cells, therefore possessing the potential for personalized therapy (Osborn et al., 2020). As we have discussed, neuroinflammation could occur prior to LB formation in grafted neurons, which may therefore facilitate the development of Lewy pathology and affect the sustainability of grafted cell function (Olanow et al., 2019). Minimizing neuroinflammation could benefit the transplantation trial by tempering the accumulation and aggregation of α-syn in grafted cells. Nevertheless, elimination of using immunosuppression could benefit the patients by decreasing the possible risks due to systematic immune system inhibition (Osborn et al., 2020). From a histological point of view, hiPSCs still face the challenges of surviving, reinnervation, and developments of Lewy pathology. As for the enhancement of survival and reinnervation, first, autologous transplantation using hiPSCs has been shown to form extensive reinnervation to the host tissue as proven by several non-human primate trials such as the proof-of-concept work published by Hallett et al. (2015), where grafted hiPSCs derived neuron successfully reinnervate and formed dense synapses with the host putamen. Without the need of immunosuppressing treatment, autologous grafting generated minimal immune responses in the CNS (Hallett et al., 2015). Second, pretreatment using neurotrophic factors such as GDNF was shown to promote DA neuron maturity through activating mitogen-activated protein kinase (MAPK) signaling pathway, therefore leading to better behavioral recovery (Gantner et al., 2020). Although it is still possible that grafted cells derived from hiPSCs will eventually go through the same pathological process as seen in fetal DA transplantation, modification and treatments of iPSCs could be performed prior to the transplantation to increase longevity of functional grafted cells. Besides the strategies to prevent α-syn aggregation as discussed above, genetic editing using CRISPR-Cas9 has been shown to resist Lewy pathology formation, where partial or complete deletion of α-syn allele in hESCs resulted in decreased formation of protein aggregates *in vitro*, upon being challenged by α-syn fibrils (Chen et al., 2019). Therefore, in a summary, autologous transplantation using hiPSCs, when optimized properly and performed under strict regulatory process, could have multiple advantages over allografts.

The gratifying thing is that clinical transplantation trials using hiPSC-derived DA neuron have already begun. In Japan, Takahashi’s laboratory has performed their first transplantation trial using clinically graded hiPSC-derived neurons in late 2018 (Takahashi, 2017, 2019, 2020; Doi et al., 2020). Schweitzer et al. (2020) published their clinical follow-up of an idiopathic PD patient receiving autologous graft derived from his own fibroblasts. Without the need for any immunosuppression, the patient showed stabilized and improved clinical symptoms 2 years postsurgery, with PET suggesting the graft survival. Moreover, many groups around the world currently have ongoing or planning trials using iPSC-based transplantation. With over two decades of efforts, several groups have generated “good manufacturing practice (GMP)” level hiPSC-derived DA neurons. Researchers aiming to use iPSC-derived DA neuron for treating PD have decided to join their efforts and share obtained evidences together for maximizing what can be learnt from trials, such as the GForce-PD, formed by groups from the United States, Japan, and Europe (Barker et al., 2017).

## Summary

Fetal DA neuron transplantation has been a promising therapeutic method for PD and accelerated the progress of regenerative medicine in PD. In this review, we have summarized postmortem studies of fetal transplantation cases, analyzing cell survival, reinnervation, and graft pathology. We discussed the possible correlation between these histological features and the graft functional outcomes. In the end, we raised several possible factors that could be considered for improving the graft survival and function. With our review, we hope to make a comprehensive summary of the transplantation field and provide references for improving future fetal DA transplantation trials, more importantly cell replacement therapy overall, from a histological point of view.

## Author Contributions

J-YL and WL written and modified the manuscript. Both authors of this work have made intellectual and substantial contributions to the manuscript and approved it for publication.

## Conflict of Interest

The authors declare that the research was conducted in the absence of any commercial or financial relationships that could be construed as a potential conflict of interest.
