# Supplementary material for: Postmortem Studies of Fetal Grafts in Parkinson’s Disease: What Lessons Have We Learned?
Source: Front Cell Dev Biol. 2021 May 13;9:666675. doi: 10.3389/fcell.2021.666675 (PMC8155361; doi:10.3389/fcell.2021.666675)
Supplement: Supplementary file 1 [file Data_Sheet_1.PDF]

# Supplementary table 1: clinical follow-ups of grafted cases.

| Case number | PD history | Follow-up time | Clinical performances | | | | | | Refs[C] |
|---|---|---|---|---|---|---|---|---|---|
| | | | Main symptoms | UPDRS[A] (on/off) | H&Y[B] (on/off) | L-dopa (mg) | "Off" time | FDOPA | |
| 1 | 10Y[D] | Baseline | Severe bradykinesia, hypophonia, tremor. | NR[F]/112 | 5 | 1200 | NR | NR | (Redmond et al., 1990) |
| | | 4M[E] | No improvements. | NR | NR | 1200 | NR | NR | (Redmond et al., 1990;Spencer et al., 1992) |
| 2 | 8Y | Baseline | Tremor, bradykinesia, rigidity. | 17/78 | 1.5/3 | 1700 | 48% | RP[G]: 0.0071<br>LP[H]: 0.0087 | (Freeman et al., 1995) |
| | | 6M | Substantially improved with less freezing. | 16.0±4/ 58±10[I] | 1.4±0.2/ 2.8±0.3 | NR | 12.1± 3.7% | RP: 0.0105<br>LP: 0.0111 | (Freeman et al., 1995) |
| | | 15M | Improved continuously. | NR /49.5 | NR | 1400 | 0 | RP: 0.0144<br>LP: 0.0140 | (Kordower et al., 1996) |
| 3 | 22Y | Baseline | Tremor, rigidity, freezing spells, falling, severe dyskinesia. | 29/118.5 | 2/4 | 275 | 53% | RP: 0.0050<br>LP: 0.0078<br>RC[J]: 0.0083<br>LC[K]: 0.0093 | (Kordower et al., 1998;Hagell and Brundin, 2001) |
| | | 6M | Functional improvements in "off period"; reduced dyskinesia. | 32/88.5 | NR | 275 | 20% | RP: 0.0104<br>LP: 0.0082<br>RC: 0.0085<br>LC: 0.0067 | |
| | | 12M | Functional improvements in "off period". | 49.5/92 | NR | 400 | 20% | RP: 0.0126<br>LP: 0.0101<br>RC: 0.0098<br>LC: 0.0081 | |
| 4 | 15±6Y | Baseline | At least two of bradykinesia, rigidity, and tremor. | 27±11/ 59±21 | NR | NR | NR | NR | (Hagell and Brundin, 2001) |
| | | 7M | NR | NR | NR | NR | NR | NR | |
| 5 | 15±6Y | Baseline | At least two of bradykinesia, rigidity, and tremor. | 27±11/ 59±21 | NR | NR | NR | NR | (Hagell and Brundin, 2001) |
| | | 1Y | Worsening. | 50 (15% decrease) | NR | NR | NR | 40±42% increase[L] | |
| | | 2-3Y | NR | 40 (33% decrease) | NR | NR | NR | 100% increase | |
| 6 | 11Y | Baseline | NR | 102/155 | 2.5/5 | 600 | 50% | RP: 0.0016<br>LP: 0.0039<br>RS: 0.0018<br>LS: 0.0025 | (Mendez et al., 2002;Mendez et al., 2005) |
| | | 3Y | L[M]: progressive improvement;<br>R[N]: worsen. | 73/84 | NR | NR | 25% | RP: 0.0051<br>LP: 0.0019<br>RS: 0.0035<br>LS: 0.0030 | |
| 7 | 15Y | Baseline | NR | 78/97 | NR | NR | 50% | RP: 0.0017<br>LP: 0.0036 | (Mendez et al., 2005) |
| | | 3Y | NR | 58/67 | NR | NR | 25% | RP: 0.0071<br>LP: 0.0052 | |
| 8 | 22Y | Baseline | Motor fluctuations and dyskinesias. | 20±12.6/ 49±10.2 | NR | 1430 ±529 | 45±10 % | NR | (Olanow et al., 2003;Kordower et al., 2008a) |
| | | 2-4Y | Decreased "off period", milder dyskinesias. | 42±5.4/ 47±3.3 | NR | 1260 ±104 | 0 | Increased value: 0.04-0.12 | |
| | | 10Y-death | Progressive deterioration, falling. | NR | NR | NR | NR | NR | (Kordower et al., 2008a) |
| 9 | 12Y | Baseline | Motor complications not controlled by medication. | 20±3.1/ 83±9.6 | NR | 800 | 35±5% | P: 0.0069<br>C: 0.0125 | (Hauser et al., 1999;Kordower et al., 2008b) |
| | | 2Y | NR | 22±6/ 56±9 | NR | 800 | 20±5% | P: 0.0111<br>C: 0.0126 | (Hauser et al., 1999) |
| | | 12Y-death | Major improvement till 12Y, deteriorate till death. | NR | NR | NR | NR | NR | (Kordower et al., 2008b) |
| 10 | 15Y | Baseline | Responded well to L-dopa. | 36/84 | NR | NR | NR | NR | (Mendez et al., 2002;Mendez et al., 2008) |
| | | 21M | Temporary moderate gait improvement. | 60/116 | NR | NR | NR | R:18% increase<br>L:3% decrease | |
| 11 | 19Y | Baseline | Responded well to L-dopa. | NR | NR | NR | NR | NR | |
| | | 3M | Improved gait and mobility, no freezing episodes, fewer dyskinesias, Shorten "off" time. | NR | NR | NR | NR | NR | |
| 12 | 14Y | Baseline | Responded well to L-dopa. | 77/102 | NR | NR | NR | NR | |
| | | 2Y | Significantly improved, worsen thereafter. | 79/89 | NR | NR | NR | R:185% increase | (Mendez et al., 2008) |

| | | | | | | | | | |
|---|---|---|---|---|---|---|---|---|---|
| | | | | | | | | L:29% decrease | |
| 13 | 12Y | Baseline | R: tremor and rigidity, progressive "on-off"; L: milder symptoms. | 42.5 | NR/3 | 700 | 50% | RP: 0.0048 LP: 0.0024 RC: 0.0076 LC: 0.0081 | (Lindvall et al., 1990a) |
| | | 1Y | R: progressive reduction of rigidity, improvement of mobility, rigidity disappeared. | 21.7 | NR | 700 | 20% | RC: 0.0082 LC: 0.0101 RP: 0.0057 LP: 0.0056 130% increase | (Lindvall et al., 1992) |
| | | 3Y | R: reduced rigidity, longer "on" periods; L: worsening, worse than right-side; difficulty in gait, lumbar back pain. | 22.5 | NR/2 | 700 | 30% | R: further reduction LP: close to normal | (Lindvall et al., 1994) |
| | | 53M | L: worsening of rigidity; slightly increased "off" time, impaired balance. | 33.6 | NR/2.5 | 700 | 28% | LP: 117% increase RP: 21% decrease | (Wenning et al., 1997) |
| | | 80/24M | Modest improvement in daily living. | Slight decrease | NR/2.5 | 800 | 29% | RP: 20% increase from 2nd graft | (Hagell et al., 1999) |
| 14 | 5Y | Baseline | L: akinesia, rigidity, "wearing off", dyskinesias. | 28.6 | NR/4 | 550 | 43.1% | NR | (Wenning et al., 1997) |
| | | 23M | Mild decrease in "off" phase, rigidity, longer duration of response to L-dopa. | 38.6 | NR | 650 | 47% | Increase LP: 33% LC: 13% | |
| | | 41/18M | Overall deterioration. | 15% increase | NR | 300 | 30% | RP: 100% RC: 200% increase | (Hagell et al., 1999) |
| 15 | 14Y | Baseline | Severe hypokinesia, rigidity, tremor, unable to walk. | NR | NR/4-5 | 1200 | 60-70% | NR | (Lindvall et al., 1990b) |
| | | 6M-death | Overall mild improvement transiently; received apomorphine infusion and DBS. | No change | NR | 1200 | No change | No significant change | |
| 16 | 9Y | Baseline | L: tremor, rigidity, during "off" phase, mild symptoms during "on" periods. | 42 | NR/3 | 450 | 60% | RC: 0.0085 LC: 0.0085 RP: 0.0040 LP: 0.0050 | (Lindvall et al., 1992) |
| | | 3Y | Rigidity almost disappeared, significant bilateral improvement. | 20 | NR/1 | 0 | 0 | RC: 0.0085 LC: 0.0085 RP: 0.0120 LP: 0.0040 | (Lindvall et al., 1994) |
| | | 6Y | Mild symptoms, no "on-off" fluctuations, minimal rigidity, moderate worsening after 6Y. | 26 | NR/2 | 0 | 0 | RC: 0.0050 LC: 0.0080 RP: 0.0150 LP: 0.0040 | (Wenning et al., 1997) |
| | | 12Y-death | Increasing rigidity, gradual loss of L-dopa response, deteriorate, no graft-induced benefits remained, increased "off" time. | NR | NR | 450 | NR | NR | (Li et al., 2016) |
| 17 | 8Y | Baseline | Tremor, gait impairment with falls. | 26/52 | 2/3 | 470 | NR | L: 0.007 R: 0.005 | (Olanow et al., 2003;Kordower et al., 2017) |
| | | Post operation | Cognitive impairment, severe hypophonia, preoperative medical regimen was reinstituted, graft-induced dyskinesias, persistent freezing, visual hallucination. | 34/NP[O] | 2-3/NP | 470 | NR | L: 0.018 R: 0.016 | |

**Supplementary table 1**: the table shows the clinical follow-ups of 17 grafted patients. The case numbers of the patients refer to the same patients in supplementary table 1 and 2. [A]: UPDRS-unified Parkinson's disease rating scale; [B]: H&Y- Hoehn and Yahr Scale; [C]: Refs-references; [D]: Y-years; [E]: M-months; [F]: NR-not reported; [G]: RP-right putamen; [H]: LP-left putamen; [I]: "±"-when the data represents the result from a grouped analysis; [J]: RC-right caudate nucleus; [K]: LC-left caudate nucleus; [L]: increased value from baseline unless specified; [M]: L-left side; [N]: R-right side; [O]: NP-not performed due to the bad condition of patients.

## References


Freeman, T.B., Olanow, C.W., Hauser, R.A., Nauert, G.M., Smith, D.A., Borlongan, C.V., Sanberg, P.R., Holt, D.A., Kordower, J.H., Vingerhoets, F.J., and Et Al. (1995). Bilateral fetal nigral transplantation into the postcommissural putamen in Parkinson's disease. *Ann Neurol* 38**,** 379-388.

Hagell, P., and Brundin, P. (2001). Cell survival and clinical outcome following intrastriatal transplantation in Parkinson disease. *J Neuropathol Exp Neurol* 60**,** 741-752.

Hagell, P., Schrag, A., Piccini, P., Jahanshahi, M., Brown, R., Rehncrona, S., Widner, H., Brundin, P., Rothwell, J.C., Odin, P., Wenning, G.K., Morrish, P., Gustavii, B., Bjorklund, A., Brooks, D.J., Marsden, C.D., Quinn, N.P., and Lindvall, O. (1999). Sequential bilateral transplantation in Parkinson's disease: effects of the second graft. *Brain* 122 ( Pt 6)**,** 1121-1132.

Hauser, R.A., Freeman, T.B., Snow, B.J., Nauert, M., Gauger, L., Kordower, J.H., and Olanow, C.W. (1999). Long-term evaluation of bilateral fetal nigral transplantation in Parkinson disease. *Arch Neurol* 56**,** 179-187.

Kordower, J.H., Chu, Y., Hauser, R.A., Freeman, T.B., and Olanow, C.W. (2008a). Lewy body-like pathology in long-term embryonic nigral transplants in Parkinson's disease. *Nat Med* 14**,** 504-506.

Kordower, J.H., Chu, Y., Hauser, R.A., Olanow, C.W., and Freeman, T.B. (2008b). Transplanted dopaminergic neurons develop PD pathologic changes: a second case report. *Mov Disord* 23**,** 2303-2306.

Kordower, J.H., Freeman, T.B., Chen, E.Y., Mufson, E.J., Sanberg, P.R., Hauser, R.A., Snow, B., and Olanow, C.W. (1998). Fetal nigral grafts survive and mediate clinical benefit in a patient with Parkinson's disease. *Mov Disord* 13**,** 383-393.

Kordower, J.H., Goetz, C.G., Chu, Y., Halliday, G.M., Nicholson, D.A., Musial, T.F., Marmion, D.J., Stoessl, A.J., Sossi, V., Freeman, T.B., and Olanow, C.W. (2017). Robust graft survival and normalized dopaminergic innervation do not obligate recovery in a Parkinson disease patient. *Ann Neurol* 81**,** 46-57.

Kordower, J.H., Rosenstein, J.M., Collier, T.J., Burke, M.A., Chen, E.Y., Li, J.M., Martel, L., Levey, A.E., Mufson, E.J., Freeman, T.B., and Olanow, C.W. (1996). Functional fetal nigral grafts in a patient with Parkinson's disease: chemoanatomic, ultrastructural, and metabolic studies. *J Comp Neurol* 370**,** 203-230.

Li, W., Englund, E., Widner, H., Mattsson, B., Van Westen, D., Latt, J., Rehncrona, S., Brundin, P., Bjorklund, A., Lindvall, O., and Li, J.Y. (2016). Extensive graft-derived dopaminergic innervation is maintained 24 years after transplantation in the degenerating parkinsonian brain. *Proc Natl Acad Sci U S A* 113**,** 6544-6549.

Lindvall, O., Brundin, P., Widner, H., Rehncrona, S., Gustavii, B., Frackowiak, R., Leenders, K.L., Sawle, G., Rothwell, J.C., Marsden, C.D., and Et Al. (1990a). Grafts of fetal dopamine neurons survive and improve motor function in Parkinson's disease. *Science* 247**,** 574-577.

Lindvall, O., Rehncrona, S., Brundin, P., Gustavii, B., Astedt, B., Widner, H., Lindholm, T., Bjorklund, A., Leenders, K.L., Rothwell, J.C., and Et Al. (1990b). Neural transplantation in Parkinson's disease: the Swedish experience. *Prog Brain Res* 82**,** 729-734.

Lindvall, O., Sawle, G., Widner, H., Rothwell, J.C., Bjorklund, A., Brooks, D., Brundin, P., Frackowiak, R., Marsden, C.D., Odin, P., and Et Al. (1994). Evidence for long-term survival and function of dopaminergic grafts in progressive Parkinson's disease. *Ann Neurol* 35**,** 172-180.

Lindvall, O., Widner, H., Rehncrona, S., Brundin, P., Odin, P., Gustavii, B., Frackowiak, R., Leenders, K.L., Sawle, G., Rothwell, J.C., and Et Al. (1992). Transplantation of fetal dopamine neurons in Parkinson's disease: one-year clinical and neurophysiological observations in two patients with putaminal implants. *Ann Neurol* 31**,** 155-165.

Mendez, I., Dagher, A., Hong, M., Gaudet, P., Weerasinghe, S., Mcalister, V., King, D., Desrosiers, J., Darvesh, S., Acorn, T., and Robertson, H. (2002). Simultaneous intrastriatal and intranigral fetal dopaminergic grafts in patients with Parkinson disease: a pilot study. Report of three cases. *J Neurosurg* 96**,** 589-596.

Mendez, I., Sanchez-Pernaute, R., Cooper, O., Vinuela, A., Ferrari, D., Bjorklund, L., Dagher, A., and Isacson, O. (2005). Cell type analysis of functional fetal dopamine cell suspension transplants in the striatum and substantia nigra of patients with Parkinson's disease. *Brain* 128**,** 1498-1510.

Mendez, I., Vinuela, A., Astradsson, A., Mukhida, K., Hallett, P., Robertson, H., Tierney, T., Holness, R., Dagher, A., Trojanowski, J.Q., and Isacson, O. (2008). Dopamine neurons implanted into people with Parkinson's disease survive without pathology for 14 years. *Nat Med* 14**,** 507-509.

Olanow, C.W., Goetz, C.G., Kordower, J.H., Stoessl, A.J., Sossi, V., Brin, M.F., Shannon, K.M., Nauert, G.M., Perl, D.P., Godbold, J., and Freeman, T.B. (2003). A double-blind controlled trial of bilateral fetal nigral transplantation in Parkinson's disease. *Ann Neurol* 54**,** 403-414.

Redmond, D.E., Jr., Leranth, C., Spencer, D.D., Robbins, R., Vollmer, T., Kim, J.H., Roth, R.H., Dwork, A.J., and Naftolin, F. (1990). Fetal neural graft survival. *Lancet* 336**,** 820-822.

Spencer, D.D., Robbins, R.J., Naftolin, F., Marek, K.L., Vollmer, T., Leranth, C., Roth, R.H., Price, L.H., Gjedde, A., Bunney, B.S., and Et Al. (1992). Unilateral transplantation of human fetal mesencephalic tissue into the caudate nucleus of patients with Parkinson's disease. *N Engl J Med* 327**,** 1541-1548.

Wenning, G.K., Odin, P., Morrish, P., Rehncrona, S., Widner, H., Brundin, P., Rothwell, J.C., Brown, R., Gustavii, B., Hagell, P., Jahanshahi, M., Sawle, G., Bjorklund, A., Brooks, D.J., Marsden, C.D., Quinn, N.P., and Lindvall, O. (1997). Short- and long-term survival and function of unilateral intrastriatal dopaminergic grafts in Parkinson's disease. *Ann Neurol* 42**,** 95-107.
