# Supplementary material for: Postmortem Studies of Fetal Grafts in Parkinson’s Disease: What Lessons Have We Learned?
Source: Front Cell Dev Biol. 2021 May 13;9:666675. doi: 10.3389/fcell.2021.666675 (PMC8155361; doi:10.3389/fcell.2021.666675)
Supplement: Supplementary file 2 [file Data_Sheet_2.PDF]

Supplementary table 2: summary of transplantation procedures

Case number	Type of trial	Number of patients in the trial	Age at grafting	Grafted materials					Grafted regions	Injection tracts	Immunosuppression	References
				Age of fetuses (weeks)	Number of fetuses	Cells	Tissue blocks	Storage				
1	O-L[A]	7	63	11	R[B]: 1	N/A[C]	$1mm^3$	79D[D] cryopreserved	RC[E]	2	A[F]: 7W[G] CsA[H]	(Redmond et al., 1990)
2	O-L	4	59	6.5-9	R: 3<br>L[I]: 4	N/A	0.75 mm^3	48H[J] in 8°C hibernation medium	BP[K]	R: 6<br>L: 6	B[L]: 2W CsA<br>A: 6M[M] CsA	(Freeman et al., 1995)
3	O-L	4	61	6.5-9	R: 4<br>L: 3	N/A	0.75 mm^3	48H in 8°C hibernation medium	BP	R: 6-8<br>L: 6-8	B: 3W CsA<br>A: 6M CsA	(Kordower et al., 1998)
4	D-B[N]	40	66	7-8	R: 2<br>L: 2	N/A	Tissue blocks	Cultured in F12 medium<4W	BP	R: 2<br>L: 2	No	(Freed et al., 2001)
5	D-B	40	68	7-8	R: 2<br>L: 2	N/A	Tissue blocks	Cultured in F12 medium<4W	BP	R: 2<br>L: 2	No	
6	O-L	3	59	6-9	R:4<br>L:3	R:4.8 x 10^6<br>L: 2.6 x 10^6	N/A	6D in 4°C low salt buffer with GDNF[O]	BN[P], BP	RN: 1<br>RP: 6<br>LN: 1<br>LP: 1	B: 2W CsA<br>A: 6M CsA	(Mendez et al., 2002)
7	O-L	8	69	6-9	R:3<br>L:2	R:3.2 x 10^6<br>L: 1.3 x 10^6	N/A	6D in 4°C low salt buffer with GDNF	BP	R: 4<br>L: 4	B: 2W CsA<br>A: 6M CsA	(Mendez et al., 2000;Mendez et al., 2005)
8	D-B	34	61	6.5-9	R:4<br>L:4	N/A	Solid pieces	8°C hibernation medium	BP	R: 8<br>L: 8	B: 2W CsA<br>A: 6M CsA	(Olanow et al., 2003;Kordower et al., 2008;Chu and Kordower, 2010)
9	O-L	6	39	6.5-9	R:4<br>L:4	N/A	0.75 mm^3	2D in 8°C hibernation medium	BP	R: 6-8<br>L: 6-8	B: 2W CsA<br>A: 6M CsA	(Hauser et al., 1999)
10	O-L	3	61	6-10	R:4<br>L:3	Cells	N/A	6D in 8°C hibernation medium	BP	R: 4<br>L: 4	B: 2W CsA<br>A: 6M CsA	(Mendez et al., 2008)
11	O-L	3	61	6-10	L:2-3	Cells	N/A	NR	LP	L: 3	B: 2W CsA<br>A: 6M CsA	
12	O-L	3	55	6-10	R:4<br>L:3	Cells	N/A	6D in 8°C hibernation medium with GDNF	BP	R: 4<br>L: 4	B: 2W CsA<br>A: 6M CsA	
13	O-L	10	49	6-9	L: 4<br>R:5	Cell suspension	N/A	3H in HBSS at RT	LP<br>RP (2nd)	L: 3<br>R: 5	B: 2D<br>A: NR[Q]<br>CsA+ AZA[R]+ PRDL[S];<br>AZA discontinued at 20M	(Lindvall et al., 1990a;Lindvall et al., 1992;Hagell et al., 1999)
14	O-L	10	43	6-8	L: 5<br>R:8	Cell suspension	N/A	3H in HBSS at RT	LP<br>LC<br>R:P+C	LP: 5<br>LC: 2<br>RP: 5<br>RC: 2	B: 2D<br>A: NR<br>CsA+ AZA+ PRDL;<br>AZA discontinued at 6M	(Wenning et al., 1997;Hagell et al., 1999)
15	O-L	10	47	8-10	L: 4	Cell suspension	N/A	4-6H	LP+C	LP: 2<br>LC: 1	B: 2D<br>A: NR<br>CsA+ AZA+ PRDL	(Lindvall et al., 1990b)
16	O-L	10	59	6-8	R:4	Cell suspension	N/A	3H in HBSS at RT	R: putamen	RP: 3	B: 2D<br>A: 64M<br>CsA+ AZA+ PRDL	(Li et al., 2016)
17	Db	34	47	6.5-9	R:4<br>L:4	N/A	Solid pieces	8°C hibernation medium	B: putamen	R: 8<br>L: 8	B: 2W CsA<br>A: 6M CsA	(Olanow et al., 2003)

Supplementary table 2: the table describe the transplantation methods of the trials, including grafted material used and immunosuppression. [A]: O-L- open-labelled; [B]: R- right side; [C]: N/A- not applicable; [D]: D- days; [E]: RC- right caudate nucleus; [F]: A- after surgery; [G]: W- weeks; [H]: CsA- cyclosporine; [I]: L-left side; [J]: H- hours; [K]: BP- both putamen; [L]: B- before surgery; [M]: M- months; [N]: D-B- double-blind placebo controlled trials; [O]: GDNF- glia derived neurotrophic factors; [P]: BN- both nigra; [Q]: NR- not reported; [R]: AZA- azathioprine; [S]: PRDL- prednisolone.

References

Chu, Y., and Kordower, J.H. (2010). Lewy body pathology in fetal grafts. *Ann N Y Acad Sci* 1184**,** 55-67.

Freed, C.R., Greene, P.E., Breeze, R.E., Tsai, W.Y., Dumouchel, W., Kao, R., Dillon, S., Winfield, H., Culver, S., Trojanowski, J.Q., Eidelberg, D., and Fahn, S. (2001). Transplantation of embryonic dopamine neurons for severe Parkinson's disease. *N Engl J Med* 344**,** 710-719.

Freeman, T.B., Olanow, C.W., Hauser, R.A., Nauert, G.M., Smith, D.A., Borlongan, C.V., Sanberg, P.R., Holt, D.A., Kordower, J.H., Vingerhoets, F.J., and Et Al. (1995). Bilateral fetal nigral transplantation into the postcommissural putamen in Parkinson's disease. *Ann Neurol* 38**,** 379-388.

Hagell, P., Schrag, A., Piccini, P., Jahanshahi, M., Brown, R., Rehncrona, S., Widner, H., Brundin, P., Rothwell, J.C., Odin, P., Wenning, G.K., Morrish, P., Gustavii, B., Bjorklund, A., Brooks, D.J., Marsden, C.D., Quinn, N.P., and Lindvall, O. (1999). Sequential bilateral transplantation in Parkinson's disease: effects of the second graft. *Brain* 122 (Pt 6)**,** 1121-1132.

Hauser, R.A., Freeman, T.B., Snow, B.J., Nauert, M., Gauger, L., Kordower, J.H., and Olanow, C.W. (1999). Long-term evaluation of bilateral fetal nigral transplantation in Parkinson disease. *Arch Neurol* 56**,** 179-187.

Kordower, J.H., Chu, Y., Hauser, R.A., Freeman, T.B., and Olanow, C.W. (2008). Lewy body-like pathology in long-term embryonic nigral transplants in Parkinson's disease. *Nat Med* 14**,** 504-506.

Kordower, J.H., Freeman, T.B., Chen, E.Y., Mufson, E.J., Sanberg, P.R., Hauser, R.A., Snow, B., and Olanow, C.W. (1998). Fetal nigral grafts survive and mediate clinical benefit in a patient with Parkinson's disease. *Mov Disord* 13**,** 383-393.

Li, W., Englund, E., Widner, H., Mattsson, B., Van Westen, D., Latt, J., Rehncrona, S., Brundin, P., Bjorklund, A., Lindvall, O., and Li, J.Y. (2016). Extensive graft-derived dopaminergic innervation is maintained 24 years after transplantation in the degenerating parkinsonian brain. *Proc Natl Acad Sci U S A* 113**,** 6544-6549.

Lindvall, O., Brundin, P., Widner, H., Rehncrona, S., Gustavii, B., Frackowiak, R., Leenders, K.L., Sawle, G., Rothwell, J.C., Marsden, C.D., and Et Al. (1990a). Grafts of fetal dopamine neurons survive and improve motor function in Parkinson's disease. *Science* 247**,** 574-577.

Lindvall, O., Rehncrona, S., Brundin, P., Gustavii, B., Astedt, B., Widner, H., Lindholm, T., Bjorklund, A., Leenders, K.L., Rothwell, J.C., and Et Al. (1990b). Neural transplantation in Parkinson's disease: the Swedish experience. *Prog Brain Res* 82**,** 729-734.

Lindvall, O., Widner, H., Rehncrona, S., Brundin, P., Odin, P., Gustavii, B., Frackowiak, R., Leenders, K.L., Sawle, G., Rothwell, J.C., and Et Al. (1992). Transplantation of fetal dopamine neurons in Parkinson's disease: one-year clinical and neurophysiological observations in two patients with putaminal implants. *Ann Neurol* 31**,** 155-165.

Mendez, I., Dagher, A., Hong, M., Gaudet, P., Weerasinghe, S., Mcalister, V., King, D., Desrosiers, J., Darvesh, S., Acorn, T., and Robertson, H. (2002). Simultaneous intrastriatal and intranigral fetal dopaminergic grafts in patients with Parkinson disease: a pilot study. Report of three cases. *J Neurosurg* 96**,** 589-596.

Mendez, I., Dagher, A., Hong, M., Hebb, A., Gaudet, P., Law, A., Weerasinghe, S., King, D., Desrosiers, J., Darvesh, S., Acorn, T., and Robertson, H. (2000). Enhancement of survival of stored dopaminergic cells and promotion of graft survival by exposure of human fetal nigral tissue to glial cell line--derived neurotrophic factor in patients with Parkinson's disease. Report of two cases and technical considerations. *J Neurosurg* 92**,** 863-869.

Mendez, I., Sanchez-Pernaute, R., Cooper, O., Vinuela, A., Ferrari, D., Bjorklund, L., Dagher, A., and Isacson, O. (2005). Cell type analysis of functional fetal dopamine cell suspension transplants in the striatum and substantia nigra of patients with Parkinson's disease. *Brain* 128**,** 1498-1510.

Mendez, I., Vinuela, A., Astradsson, A., Mukhida, K., Hallett, P., Robertson, H., Tierney, T., Holness, R., Dagher, A., Trojanowski, J.Q., and Isacson, O. (2008). Dopamine neurons implanted into people with Parkinson's disease survive without pathology for 14 years. *Nat Med* 14**,** 507-509.

Olanow, C.W., Goetz, C.G., Kordower, J.H., Stoessl, A.J., Sossi, V., Brin, M.F., Shannon, K.M., Nauert, G.M., Perl, D.P., Godbold, J., and Freeman, T.B. (2003). A double-blind controlled trial of bilateral fetal nigral transplantation in Parkinson's disease. *Ann Neurol* 54**,** 403-414.

Redmond, D.E., Jr., Leranth, C., Spencer, D.D., Robbins, R., Vollmer, T., Kim, J.H., Roth, R.H., Dwork, A.J., and Naftolin, F. (1990). Fetal neural graft survival. *Lancet* 336**,** 820-822.

Wenning, G.K., Odin, P., Morrish, P., Rehncrona, S., Widner, H., Brundin, P., Rothwell, J.C., Brown, R., Gustavii, B., Hagell, P., Jahanshahi, M., Sawle, G., Bjorklund, A., Brooks, D.J., Marsden, C.D., Quinn, N.P., and Lindvall, O. (1997). Short- and long-term survival and function of unilateral intrastriatal dopaminergic grafts in Parkinson's disease. *Ann Neurol* 42**,** 95-107.
