# Supplementary material for: Postmortem Studies of Fetal Grafts in Parkinson’s Disease: What Lessons Have We Learned?
Source: Front Cell Dev Biol. 2021 May 13;9:666675. doi: 10.3389/fcell.2021.666675 (PMC8155361; doi:10.3389/fcell.2021.666675)
Supplement: Supplementary file 3 [file Data_Sheet_3.PDF]

## Supplementary table 3: graft survival, reinnervation and pathology.

| Case number | Cause of death | Age of grafts | Postmortem interval | Fixation | Survival of grafted neurons | | Reinnervation | | | Graft pathology | | | Host Lewy pathology | References |
|---|---|---|---|---|---|---|---|---|---|---|---|---|---|---|
| | | | | | Number of TH+ neurons | Functional profiles of grafted cells | Area | Pattern | TH+ fiber | Diffused α-syn | LBs[E] | Neuroinflammation | | |
| 1 | Complication of Parkinsonism | 4M[A] | 4-5H[B] | NR[C] | 0 | NM+[D] | No reinnervation | NR | NR | NR | NR | NR | NR | (Hagell and Brundin, 2001) |
| 2 | Pulmonary embolism | 18M | <4H | Zamboni's fixative | R[F]: 126168<br>L[G]: 81905 | NM+: present;<br>DAT+[H]: similar to TH+;<br>GAD+[I]: very few;<br>CytC+[J]: comparable to normal. | RP[K]: 53%;<br>LP: 23%;<br>RC[L]: innervated. | Patch-matrix; synapse formation | Fiber outgrowth:<br>L: 2-3mm;<br>R: 5-7mm;<br>2-3 folds of NG[M]. | Not detectable | NR | Numerous immune cell infiltration | NR | (Freeman et al., 1995;Kordower et al., 1996;Hauser et al., 1999;Hagell and Brundin, 2001) |
| 3 | Cardiac arrhythmia | 19M | 5H | Zamboni's fixative, cryopreserved | T[N]: 118258 | NR | P: 78% | Patch-matrix; synapse formation | Thick fibers | Not detectable | NR | Numerous immune cell infiltration | NR | (Kordower et al., 1998;Hagell and Brundin, 2001;Chu and Kordower, 2010) |
| 4 | Automobile accidents | 7M | NR | NR | R: 34115<br>L: 38392 | NM+: absent | NR | NR | Fiber outgrowth: 2-3mm | NR | Not detectable | CD3+, HLAII+[O] present. | SN[P]: pigmented DA[Q] neurons with LBs | (Freed et al., 2001) |
| 5 | Myocardial infarction | 3Y | NR | NR | R: 36796<br>L: 6840 | NM+: present | P: 100% | Complete | NR | NR | Not detectable | CD3+, HLAII+ present. | SN: pigmented DA neurons with LBs | (Freed et al., 2001) |
| 6 | Acute renal failure | 4Y4M | 4-5H | Ice-cold PFA[R] buffer | RP: 202933<br>RN[S]: 4289<br>L:0 | NM+: present;<br>RP: 26% CB+[T], 68% Girk2+[U];<br>RN: 49% CB+, 48% Girk2+;<br>DAT+: intense punctate;<br>Tom20+: similar to normal. | RP: 100%;<br>RC: innervated;<br>L: no. | Complete | R: density similar to normal;<br>L: none | NR | Absence of LBs | Minor CD45+ increase | NR | (Mendez et al., 2002;Mendez et al., 2005;Hallett et al., 2014) |
| 7 | Myocardial infarct | 3Y8M | 4-5H | Ice-cold PFA buffer | R: 127189<br>L: 98913 | NM+: present;<br>R: 47% CB+, 71% Girk2+;<br>L: 48%CB+, 67% Girk2+;<br>DAT+: intense punctate;<br>Tom20+: similar to normal. | B: to caudate putamen | Partial | Similar density to normal | NR | Absence of LBs | No activation of microglia or astroglia | SN: presence of LBs | (Mendez et al., 2000a;Mendez et al., 2000b;Mendez et al., 2005;Hallett et al., 2014) |
| 8 | Cardiac arrest | 14Y | 11H | Zamboni's fixative | Robust graft survival | NM+: present;<br>VMAT2+[V]: similar to TH+;<br>DAT+: light to no staining<br>Girk2+: present. | NR | Patch-matrix<br>Synapse formation | NR | Increased cytoplasmic α-syn | Aggregated masses and neurites: α-syn+, ubiquitin+, S129+[W]. | Filled with activated microglia, CD45+, systematic inflammation | SN: loss of DA neurons; ubiquitin+ LBs. | (Kordower et al., 2008a;Chu and Kordower, 2010) |
| 9 | NR | 14Y | 3H | Zamboni's fixative | Robust graft survival<br>R<L | NM+: present;<br>VMAT2+: present;<br>DAT+: little | B: extensive through putamen | Organotype | Large amount of fibers | NR | Large intracellular inclusions, α-syn+, ubiquitin+, S129+; rare LNs[X] | Numerous CD45+ cells, systematic inflammation | SN: LBs present | (Kordower et al., 2008b;Chu and Kordower, 2010) |
| 10 | Myocardial infarct | 9Y | 3–4H | Ice-cold PFA buffer | R: 11687<br>L: 11100 | Girk2+: host graft interface;<br>CB+: center;<br>Serotonin+: robust punctate expression;<br>DAT+: decreased;<br>Tom20+: no accumulation. | NR | Well-integrated | Extensive neuritic outgrowth | NR | Absence of LBs | No major immune reaction | SN: ubiquitin+ LBs;<br>LBs found in upper raphe nucleus, neocortex and putamen;<br>Tom20 accumulation. | (Mendez et al., 2008;Hallett et al., 2014) |
| 11 | Myocardial infarct | 14Y | 3–4H | Ice-cold PFA buffer | L: 9861 | | | | | | | | | |
| 12 | Renal failure | 9Y | 3–4H | Ice-cold PFA buffer | R: 21552<br>L: 10917 | | | | | | | | | |
| 13 | Cardiac arrest due to advanced PD | R:12Y<br>L:16Y | NR | 6% PFA for 2 months | Per tract<br>L: 12100–29500<br>R: 14400–27600 | NM+: present;<br>Girk2+: 27%;<br>CB+: 38%;<br>myelinated axons. | Entire host striatum | Partial | Long processes, forming dense networks | Cells contain α-syn:<br>R:40%<br>L:80% | LBs: α-syn+, ubiquitin+, S129+;<br>LNs: present;<br>Tau+: punctate;<br>LBs in grafted cells:<br>R: 2%<br>L: 5% | No strong activation; astrogliosis present. | SN: loss of DA neurons;<br>21% of NM+ neuron contain ubiquitin+ LBs;<br>cortex: LBs present. | (Kordower et al., 2008a;Li et al., 2010;Kurowska et al., 2011) |
| 14 | Aspiration pneumonia | R:11Y<br>L:13Y | NR | L: flash frozen. R: fixed in 10% formalin | Survival of TH cells | NR | In the grafts and surrounding striatum | Partial | Long processes, forming dense networks | NR | LBs: α-syn+, ubiquitin+, S129+;<br>LNs: present. | | | |
| 15 | Acute bronchopneumonia | 22Y | NR | 6% PFA for 2 months | 2700 in one tract | NM+: present;<br>DAT+: weak, 'patchy';<br>atrophic neurons | Rarely crossed the graft-host boundary | Almost no | Few fibers | Cells contain α-syn: 50% | LBs in grafted cells: 1.2%; ThS+[Y], S129+ | Few activated GFAP+[Z] or IBA1+[a] cell | 7% only NM+ | (Kurowska et al., 2011) |
| 16 | Cardiac insufficiency | 24Y | 4D[b] | 6% PFA for 1 month, transferred to 20% sucrose solution | T: 42928 | NM+: without TH: 24%;<br>VMAT2+: weak;<br>DAT+: weak. | Entire right putamen | Complete | Rich network; density comparable to normal. | NR | LBs in grafted cells: 12%; ubiquitin+, S129+ | No evidence of an ongoing inflammatory response | General atrophy; widening of ventricles; extensive Lewy pathology in all brain regions;<br>Braak stage 6;<br>SN: 20% of NM+ neuron contain LBs. | (Li et al., 2016) |
| 17 | Palliative care | 16Y | 6H | Zamboni's fixative | L: 332022<br>R: 327585 | NM+ without TH:<br>L:43,321, R:34,249. | Bilateral entire putamen | Patch-matrix | Density indistinguishable from normal; synaptic contacts; myelinated putative graft-derived axons. | NR | LBs in grafted cells:<br>R:10.7%; L: 27%;<br>ThS+, S129+. | Density of GFAP and IBA1 was similar between the graft and host. | SN: loss of DA neurons and ubiquitin+ LBs;<br>P: Lewy pathology;<br>cortex: β-amyloid deposition;<br>Meynert: loss of cholinergic neurons;<br>raphe:loss of serotonergic neurons;<br>locus coeruleus: loss of NA[c] cells. | (Kordower et al., 2017) |

**Supplementary table 3:** the table lists information regarding the grafted cell survival, reinnervation and pathology in the grafts and host brains. [A]: M- months; [B]: H- hours; [C]: NR- not reported; [D]: NM+- neuromelanin positive; [E]: LBs- Lewy bodies; [F]: R- right side; [G]: L- left side; [H]: DAT- dopamine transporter; [I]: GAD- glutamic acid decarboxylase; [J]: CytC- cytochrome C; [K]: P- putamen; [L]: C- caudate nucleus; [M]: NG- non-grafted side; [N]: T- total; [O]: HLA- human leukocyte antigen gene complex; [P]: SN- substantia nigra; [Q]: DA- dopaminergic; [R]: PFA- Paraformaldehyde; [S]: RN- right nigra; [T]: CB- calbindin; [U]: Girk2- G protein-activated inward rectifier potassium channel 2; [V]: VMAT2- vesicular monoamine transporter 2; [W]: S129- phosphorylated α-syn 129; [X]: LNs- Lewy neurites; [Y]: ThS- thioflavin S; [Z]: GFAP- Glial fibrillary acidic protein; [a]: IBA1- Allograft inflammatory factor 1; [b]: D- days; [c]: NA- noradrenalin.

### References


Chu, Y., and Kordower, J.H. (2010). Lewy body pathology in fetal grafts. *Ann N Y Acad Sci* 1184**,** 55-67.

Freed, C.R., Greene, P.E., Breeze, R.E., Tsai, W.Y., Dumouchel, W., Kao, R., Dillon, S., Winfield, H., Culver, S., Trojanowski, J.Q., Eidelberg, D., and Fahn, S. (2001). Transplantation of embryonic dopamine neurons for severe Parkinson's disease. *N Engl J Med* 344**,** 710-719.

Freeman, T.B., Olanow, C.W., Hauser, R.A., Nauert, G.M., Smith, D.A., Borlongan, C.V., Sanberg, P.R., Holt, D.A., Kordower, J.H., Vingerhoets, F.J., and Et Al. (1995). Bilateral fetal nigral transplantation into the postcommissural putamen in Parkinson's disease. *Ann Neurol* 38**,** 379-388.

Hagell, P., and Brundin, P. (2001). Cell survival and clinical outcome following intrastriatal transplantation in Parkinson disease. *J Neuropathol Exp Neurol* 60**,** 741-752.

Hallett, P.J., Cooper, O., Sadi, D., Robertson, H., Mendez, I., and Isacson, O. (2014). Long-term health of dopaminergic neuron transplants in Parkinson's disease patients. *Cell Rep* 7**,** 1755-1761.

Hauser, R.A., Freeman, T.B., Snow, B.J., Nauert, M., Gauger, L., Kordower, J.H., and Olanow, C.W. (1999). Long-term evaluation of bilateral fetal nigral transplantation in Parkinson disease. *Arch Neurol* 56**,** 179-187.

Kordower, J.H., Chu, Y., Hauser, R.A., Freeman, T.B., and Olanow, C.W. (2008a). Lewy body-like pathology in long-term embryonic nigral transplants in Parkinson's disease. *Nat Med* 14**,** 504-506.

Kordower, J.H., Chu, Y., Hauser, R.A., Olanow, C.W., and Freeman, T.B. (2008b). Transplanted dopaminergic neurons develop PD pathologic changes: a second case report. *Mov Disord* 23**,** 2303-2306.

Kordower, J.H., Freeman, T.B., Chen, E.Y., Mufson, E.J., Sanberg, P.R., Hauser, R.A., Snow, B., and Olanow, C.W. (1998). Fetal nigral grafts survive and mediate clinical benefit in a patient with Parkinson's disease. *Mov Disord* 13**,** 383-393.

Kordower, J.H., Goetz, C.G., Chu, Y., Halliday, G.M., Nicholson, D.A., Musial, T.F., Marmion, D.J., Stoessl, A.J., Sossi, V., Freeman, T.B., and Olanow, C.W. (2017). Robust graft survival and normalized dopaminergic innervation do not obligate recovery in a Parkinson disease patient. *Ann Neurol* 81**,** 46-57.

Kordower, J.H., Rosenstein, J.M., Collier, T.J., Burke, M.A., Chen, E.Y., Li, J.M., Martel, L., Levey, A.E., Mufson, E.J., Freeman, T.B., and Olanow, C.W. (1996). Functional fetal nigral grafts in a patient with Parkinson's disease: chemoanatomic, ultrastructural, and metabolic studies. *J Comp Neurol* 370**,** 203-230.

Kurowska, Z., Englund, E., Widner, H., Lindvall, O., Li, J.Y., and Brundin, P. (2011). Signs of degeneration in 12-22-year old grafts of mesencephalic dopamine neurons in patients with Parkinson's disease. *J Parkinsons Dis* 1**,** 83-92.

Li, J.Y., Englund, E., Widner, H., Rehncrona, S., Bjorklund, A., Lindvall, O., and Brundin, P. (2010). Characterization of Lewy body pathology in 12- and 16-year-old intrastriatal mesencephalic grafts surviving in a patient with Parkinson's disease. *Mov Disord* 25**,** 1091-1096.

Li, W., Englund, E., Widner, H., Mattsson, B., Van Westen, D., Latt, J., Rehncrona, S., Brundin, P., Bjorklund, A., Lindvall, O., and Li, J.Y. (2016). Extensive graft-derived dopaminergic innervation is maintained 24 years after transplantation in the degenerating parkinsonian brain. *Proc Natl Acad Sci U S A* 113**,** 6544-6549.

Mendez, I., Dagher, A., Hong, M., Gaudet, P., Weerasinghe, S., Mcalister, V., King, D., Desrosiers, J., Darvesh, S., Acorn, T., and Robertson, H. (2002). Simultaneous intrastriatal and intranigral fetal dopaminergic grafts in patients with Parkinson disease: a pilot study. Report of three cases. *J Neurosurg* 96**,** 589-596.

Mendez, I., Dagher, A., Hong, M., Hebb, A., Gaudet, P., Law, A., Weerasinghe, S., King, D., Desrosiers, J., Darvesh, S., Acorn, T., and Robertson, H. (2000a). Enhancement of survival of stored dopaminergic cells and promotion of graft survival by exposure of human fetal nigral tissue to glial cell line--derived neurotrophic factor in patients with Parkinson's disease. Report of two cases and technical considerations. *J Neurosurg* 92**,** 863-869.

Mendez, I., Hong, M., Smith, S., Dagher, A., and Desrosiers, J. (2000b). Neural transplantation cannula and microinjector system: experimental and clinical experience. Technical note. *J Neurosurg* 92**,** 493-499.

Mendez, I., Sanchez-Pernaute, R., Cooper, O., Vinuela, A., Ferrari, D., Bjorklund, L., Dagher, A., and Isacson, O. (2005). Cell type analysis of functional fetal dopamine cell suspension transplants in the striatum and substantia nigra of patients with Parkinson's disease. *Brain* 128**,** 1498-1510.

Mendez, I., Vinuela, A., Astradsson, A., Mukhida, K., Hallett, P., Robertson, H., Tierney, T., Holness, R., Dagher, A., Trojanowski, J.Q., and Isacson, O. (2008). Dopamine neurons implanted into people with Parkinson's disease survive without pathology for 14 years. *Nat Med* 14**,** 507-509.
